# Rapid Antigen Test for Postmortem Evaluation of SARS-CoV-2 Carriage

**DOI:** 10.3201/eid2706.210226

**Published:** 2021-06

**Authors:** Martin Zacharias, Verena Stangl, Andrea Thüringer, Martina Loibner, Philipp Wurm, Stella Wolfgruber, Kurt Zatloukal, Karl Kashofer, Gregor Gorkiewicz

**Affiliations:** Medical University of Graz, Graz, Austria

**Keywords:** SARS-CoV-2, COVID-19, coronavirus, 2019 novel coronavirus disease, severe acute respiratory syndrome coronavirus 2, zoonoses, coronavirus disease, viruses, autopsy, rapid antigen test, PCR, virus cultivation

## Abstract

Detecting severe acute respiratory syndrome coronavirus 2 in deceased patients is key when considering appropriate safety measures to prevent infection during postmortem examinations. A prospective cohort study comparing a rapid antigen test with quantitative reverse transcription PCR showed the rapid test’s usability as a tool to guide autopsy practice.

Rapid detection of severe acute respiratory syndrome coronavirus 2 (SARS-CoV-2) is essential to prevent viral dissemination. Rapid antigen tests (RATs) have recently been approved and are now widely used in the current coronavirus disease (COVID-19) pandemic (*1*). Although the performance of RATs has been evaluated extensively in clinics (*2*–*4*), data on postmortem testing are still lacking (*5*).

We performed a prospective cohort study in which we evaluated the performance of the Roche/SD Biosensor SARS-CoV-2 RAT (https://www.roche.com*)* in 30 consecutive deceased COVID-19 patients at the University Hospital, Medical University of Graz (Graz, Austria), during November 28–December 23, 2020. We tested each corpse with nasopharyngeal swabs for RAT (using the manufacturer’s kit) and eSwabs (https://www.copanusa.com) for quantitative reverse transcription PCR (qRT-PCR) targeted to the viral envelope (E) and nucleocapsid (N) genes of SARS-CoV-2. Furthermore, we used virus isolation from lung tissue swabs from an additional cohort of deceased COVID-19 patients (n = 11) to compare molecular detection and virus cultivability (Appendix).

All patients were Caucasian, median age was 78 years (range 62–93 years), and 51.2% were female. The median disease duration (interval between the first positive SARS-CoV-2 PCR and death) was 11 days (range 1–43 days). The median postmortem interval (time between death and specimen sampling) was 23 hours (range 8–124 hours; Table; Appendix).

**Table:** Patient characteristics and postmortem data for investigation of rapid antigen test for postmortem evaluation of SARS-CoV-2 carriage, Graz, Austria*

Characteristic	RAT cohort, n = 30	Culture cohort, n = 11
Age, y, median (range)	78 (62–93)	79 (65–93)
Sex, no. (%)		
M	14 (47.7)	6 (56)
F	16 (53.3)	5 (45.4)
Disease duration,† d, median (range)	12 (1–43)	9 (3–34)
Postmortem interval‡, h, median (range)	22 (8–124)	25 (14–68)
qRT-PCR positive, no. (%)	24 (80)	11 (100)
C_t_ value, median (range)		
E gene	22.8 (14.1–37.3)	19.9 (13.7–36.0)
N gene	26.9 (18.0–34.6)	24.6 (17.3–33.7)
Cultivation positive, no. (%)	NA	7 (63.6)
RAT positive, no. (%)	17 (56.7%)	NA
Total RAT specificity (95% CI﻿§), n = 30	100% (61%–100%)	NA
RAT sensitivity (95% CI﻿§), n = 30	70.8% (50.8%–85.1%)	NA
Total, n = 30		
C_t_ <35,¶ n = 23	73.9% (53.5%–87.5%)	NA
C_t_ <30,¶ n = 18	94.4% (74.2%–99.7%)	NA
C_t_ <25,¶ n = 16	100% (80.6%–100%)	NA

*C_t_, cycle threshold; E, envelope; N, nucleocapsid; NA, not applicable; qRT-PCR, quantitative reverse transcription PCR: RAT, rapid antigen test; SARS-CoV-2, severe acute respiratory syndrome coronavirus 2. †Interval from first positive (antemortem) SARS-CoV-2 PCR to death. ‡Interval from death to specimen sampling. ﻿§Determined via the hybrid Wilson/Brown method (*10*). ¶Determined via E gene qRT-PCR.

PCR is the current standard for SARS-CoV-2 detection (*1*,*2*). In our cohort, qRT-PCR targeted to the E gene showed a higher sensitivity than qRT-PCR for the N gene (Appendix Figure 1). Consequently, we used E gene qRT-PCR as the reference in subsequent evaluations. Results showed that 80% (24/30) of cases were qRT-PCR positive, whereas 56.7% (17/30) were RAT positive (Figure, panel A). RAT had an overall specificity of 100% (95% CI 61%–100%) and an overall sensitivity of 70.8% (95% CI 50.8%–85.1%) when using E gene qRT-PCR as the reference. RAT negative cases showed significantly higher C_t_ values in qRT-PCR compared with RAT positive cases (mean 38.24 [SD 7.01] vs 20.74 [SD 3.46]; Figure, panel B). Correspondingly, RAT sensitivity increased when cases were stratified according to C_t_ values (C_t_ <35, sensitivity 73.9% [95% CI 53.5%–87.5%]; C_t_ <30, sensitivity 94.4% [95% CI 74.2%–99.7%]; C_t_ <25, sensitivity 100% [95% CI 80.6%–100%]) (Table; Appendix Table 1). Furthermore, when we compared qRT-PCR results from nasopharyngeal swabs of patients in which viral culture was performed (from corresponding lung tissue swabs of an additional cohort), cultivability was restricted to cases with C_t_ values <23.7, which is below the threshold of false-negative RAT cases (C_t_ values >25.8; Figure, panels B, C). These results are in line with most clinical RAT studies that also used virus culture (*2*–*4*,*6*), in which cultivability is exceedingly rare in cases with low viral loads determined with qRT-PCR. We used cultivation from lung tissue swab specimens for this analysis because the lung often shows increased SARS-CoV-2 loads in deceased patients (*7*; Appendix Table 2) and therefore represents a major infection source during autopsy.

**Figure:**
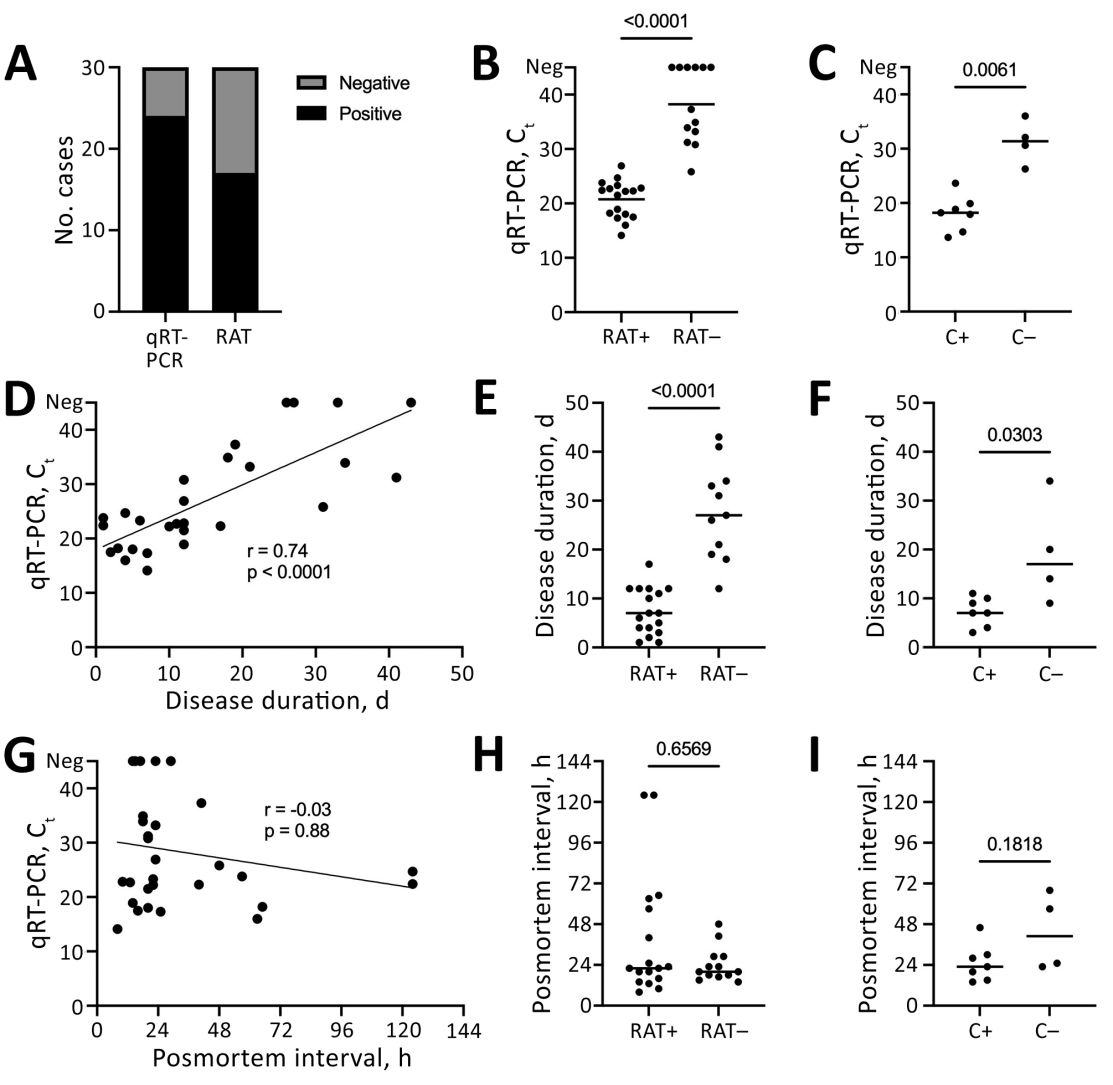
Postmortem detection and cultivation of SARS-CoV-2 for investigation of RAT for postmortem evaluation of SARS-CoV-2 carriage, Graz, Austria. A) Among 30 deceased SARS-CoV-2 patients, RAT detected fewer positive cases than did qRT-PCR. B) RAT-negative cases show significantly higher C_t_ values in qRT-PCR compared with RAT-positive cases (Mann-Whitney test). C) Cultivation negative and positive cases mirror C_t_ values of RAT results (Mann-Whitney test). D–F) Longer disease durations are significantly correlated with higher C_t_ values (Spearman correlation test; D), negative RAT results (Mann-Whitney test; E), and negative cultivation results (Mann-Whitney test; F). G–I) No significant correlation was found between postmortem intervals and C_t_ values (Spearman correlation test; G), RAT results (Mann-Whitney test; H), or cultivation results (Mann-Whitney test; I). C, cultivation; C_t_, cycle threshold; neg, negative; qRT-PCR, quantitative reverse transcription PCR; RAT, rapid antigen test; SARS-CoV-2, severe acute respiratory syndrome coronavirus 2; +, positive; –, negative.

Furthermore, we determined parameters that influenced test performance. We noted a significant positive correlation between disease duration and C_t_ values (Figure, panel D). Such correlation was also evident in RATs; all cases with disease courses >17 days were RAT negative (Figure, panel E). Postmortem intervals did not correlate with C_t_ values or RAT results (Figure, panels G, H). Thus, a long disease duration rather than a long postmortem interval seems to be the main factor for increased C_t_ values and negative RATs. RAT and cultivation results closely mirrored each other with respect to viral load (Figure, panels B, C), disease duration (Figure, panels E, F), and postmortem interval (Figure, panels H, I).

Although RAT had an overall lower sensitivity than qRT-PCR in this study, our data suggest that viral loads of false-negative RAT cases are probably below the threshold of cultivability. Because culture is regarded as a measure of virus viability and infectivity (*8*), these cases likely pose only minimal risks of SARS-CoV-2 transmission during postmortem examinations. However, each corpse having a postmortem evaluation must be treated as potentially infectious. Even a PCR-negative nasopharyngeal swab specimen does not exclude the presence of viable virus in other body sites, as shown in COVID-19 (*7*), thus emphasizing the general application of appropriate autopsy safety measures. 

In conclusion, RAT should not be seen as a potential replacement for but rather as an addition to current postmortem testing strategies. Especially when qRT-PCR is not readily available, RAT might be useful in selecting the most hazardous corpses that should be examined under special conditions (e.g., Biosafety Level 3 [*9*]). RAT could therefore be a valuable adjunct tool in guiding autopsy practice.

Appendix Additional information on materials and methods involved in postmortem detection and cultivation of SARS-CoV-2.
