# Supplementary material for: Rapid Antigen Test for Postmortem Evaluation of SARS-CoV-2 Carriage
Source: Emerg Infect Dis. 2021 Jun;27(6):1734–7. doi: 10.3201/eid2706.210226 (PMC8153888; doi:10.3201/eid2706.210226)
Supplement: Appendix — Additional information on materials and methods involved in postmortem detection and cultivation of SARS-CoV-2. [file 21-0226-Techapp-s1.pdf]

Article DOI: https://doi.org/10.3201/eid2706.210226

Rapid Antigen Test for Postmortem Evaluation of SARS-CoV-2 Carriage

Appendix

Materials and Methods

Case Description

Clinical parameters were obtained from electronic medical records and are shown in Table 1 and Appendix Tables 1 and 2. The study was approved by the institutional review board of the Medical University of Graz, Austria (32–362ex19/20).

SARS-CoV-2 Quantitative Reverse Transcription PCR (qRT-PCR)

We extracted RNA from 200 µL eSwab solution using the Maxwell simplyRNA Blood Kit (Promega, https://www.promega.com) eluting RNA in 50 µL distilled water. qRT-PCR detected regions of the viral envelope (E) and nucleocapsid (N) specific to SARS-CoV-2 (*1*). Primers, probes, and 5 µL of RNA solution were added to 10 µL of SuperScript III One-Step RT-PCR System withµ Platinum *Taq* High Fidelity DNA Polymerase (Thermo Fisher, https://www.thermofisher.com) master mix. PCR was performed on a Quantstudio 7 instrument (Thermo Fisher) with the following cycling conditions: 55°C 15 min, 95°C 3 min; 45 cycles (95°C 15 sec; 58°C 30 sec). We used human glyceraldehyde 3-phosphate dehydrogenase (GAPDH) mRNA as internal RNA control with the same cycling conditions. All primers and probes were from Eurofins Scientific (https://www.eurofins.com).

We downloaded and processed amplification data using the qpcR package of the R project (https://www.r-project.org). Amplification efficiency plots were visually inspected, and Cp2D (cycle peak of second derivative) values were calculated for samples with valid amplification curves. We generated plots with R using the reshape, tidyverse, and ggplot packages.

SARS-CoV-2 Cultivation

For SARS-CoV-2 cultivation, we used swabs from lung parenchyma collected during autopsy. Samples were frozen (−80°C) and thawed (37°C) twice to increase cell lysis and viral release. We added 2 mL OptiPro SFM medium (GIBCO, https://www.thermofisher.com) with 4 mM L-glutamine (GIBCO) and 1% penicillin–streptomycin (10,000 U/mL; GIBCO) were added to the samples. After centrifugation (10 min, 1,500 rcf) the supernatants were filtered through a 0.45 μm membrane filter (Millipore, https://www.sigmaaldrich.com) and inoculated on Vero-E6 cells with OptiPro SFM medium with 4 mM L-glutamine and 1% penicillin–streptomycin in T25 flasks (Thermo Fisher). After 3–4 days incubation at 37°C and 5% CO_2, the whole cells were detached and passaged, including the supernatant, to new Vero-E6 cells growing in T75 flasks (Thermo Fisher). After 1 week, we harvested the cells and stored the supernatants after centrifugation (10 min, 1,500 rcf) at −80°C.

Reference

1. Corman VM, Landt O, Kaiser M, Molenkamp R, Meijer A, Chu DK, et al. Detection of 2019 novel coronavirus (2019-nCoV) by real-time RT-PCR. Euro Surveill. 2020;25:2000045. PubMed https://doi.org/10.2807/1560-7917.ES.2020.25.3.2000045

Appendix Table 1. Case characteristics and postmortem data of the RAT cohort*

Case no.	Age, y/sex	Disease duration, d†	Postmortem interval, h‡	RAT	qRT-PCR	C_t value E gene	C_t value N gene	C_t value GAPDH
1	79/F	5	20	Positive	Positive	18	22	21.3
2	75/F	31	48	Negative	Positive	25.8	30.3	25.9
3	72/F	1	124	Positive	Positive	22.4	27.7	26.4
4	71/F	18	18	Negative	Positive	34.9	34.6	22.9
5	89/F	12	20	Negative	Positive	30.8	33.2	19.8
6	73/F	6	22	Positive	Positive	23.3	28	22.2
7	88/M	11	13	Positive	Positive	22.7	27.4	25.4
8	87/M	NA	14	Negative	Negative	NA	NA	21.2
9	73/M	41	20	Negative	Positive	31.2	33.2	23.8
10	78/M	17	40	Positive	Positive	22.3	26.9	26
11	87/F	4	63	Positive	Positive	16	20.8	25.7
12	70/M	3	65	Positive	Positive	18.2	22.6	22.6
13	84/F	1	57	Positive	Positive	23.8	28.1	27.5
14	90/F	19	41	Negative	Positive	37.3	NA	24.2
15	76/M	27	29	Negative	Negative	NA	NA	23.5
16	78/F	7	25	Positive	Positive	17.3	22.2	21.9
17	76/M	12	14	Positive	Positive	18.9	23.5	25.1
18	62/M	34	18	Negative	Positive	33.9	NA	23.2
19	90/F	4	124	Positive	Positive	24.7	29.2	26.5
20	67/M	12	23	Positive	Positive	26.9	29.7	26.7
21	73/F	10	22	Positive	Positive	22.2	25.7	23.5
22	73/F	12	20	Positive	Positive	21.5	24.4	20.8
23	80/F	12	10	Positive	Positive	22.8	25.9	25.3
24	77/M	43	15	Negative	Negative	NA	NA	25.7
25	93/F	2	16	Positive	Positive	17.5	20.8	23.6
26	87/M	26	29	Negative	Negative	NA	NA	24.2
27	91/M	21	23	Negative	Positive	33.2	NA	23.5
28	77/F	NA	23	Negative	Negative	NA	NA	21
29	79/M	33	17	Negative	Negative	NA	NA	29.7
30	87/M	7	8	Positive	Positive	14.1	18	25.4

*C_t, cycle threshold; E, envelope; GAPDH, human glyceraldehyde 3-phosphate dehydrogenase; N, nucleocapsid; NA, not applicable; RAT, rapid antigen test.
†Interval from first positive (antemortem) SARS-CoV-2 PCR to death.
‡interval from death to specimen sampling.

Appendix Table 2. Case characteristics and postmortem data of the cultivation cohort*

Case no.	Age, y/sex	Disease duration, d†	Postmortem interval, h‡	Cultivation	qRT-PCR	C_t (E gene) nasopharynx	C_t (E gene) lung	C_t (N gene) nasopharynx	C_t (N gene) lung
1	78/M	10	28	Positive	Positive	23.7	21.3	29.7	27.7
2	82/M	7	15	Positive	Positive	18.2	17.2	25.3	NP
3	78/M	9	68	Negative	Positive	32.1	32.1	33.7	33.3
4	92/F	3	14	Positive	Positive	17.9	16.6	21.5	20
5	71/F	4	30	Positive	Positive	14.7	14.5	18.4	19.5
6	93/F	9	20	Positive	Positive	13.7	16.5	17.3	21.1
7	79/M	14	25	Negative	Positive	26.3	29.5	30	32.8
8	67/M	20	23	Negative	Positive	36	34.4	NA	NA
9	80/F	7	46	Positive	Positive	18.9	22.5	22.7	26
10	80/F	11	23	Positive	Positive	19.9	25.5	23.9	28.5
11	65/M	34	57	Negative	Positive	30.6	NA	32.4	NA

* C_t, cycle threshold; E, envelope; N, nucleocapsid; NA, not applicable; NP, not performed; RAT, rapid antigen test.
†Interval from first positive (antemortem) SARS-CoV-2 PCR to death.
‡Interval from death to specimen sampling.

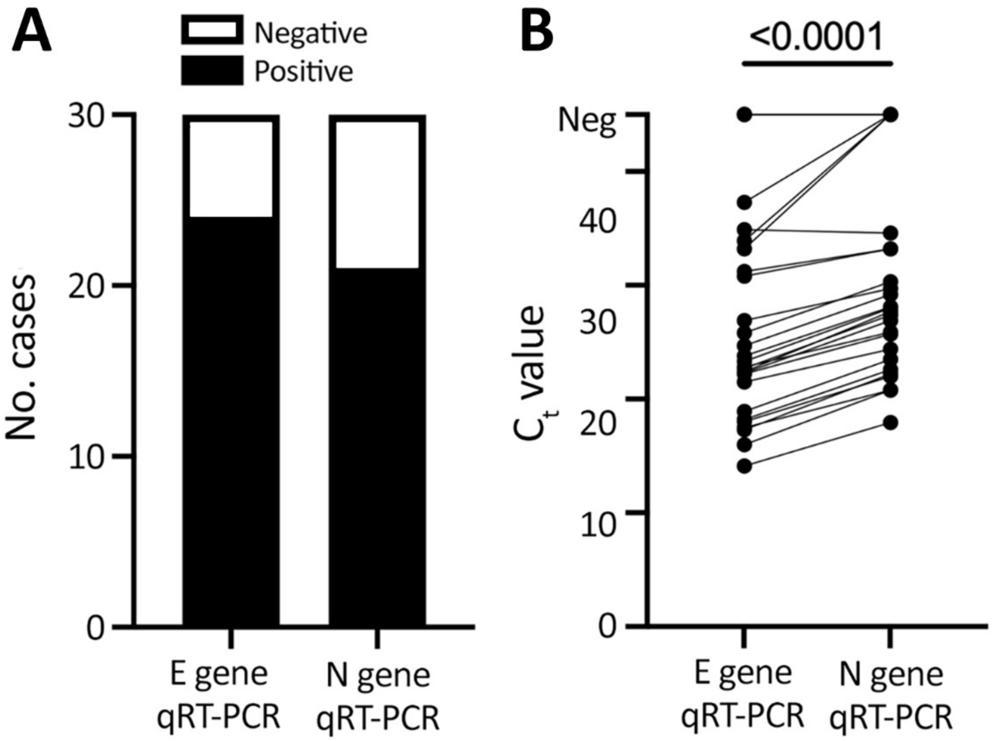

Appendix Figure 1. E gene qRT-PCR has a higher sensitivity than N gene qRT-PCR. A) More negative cases in N gene qRT-PCR compared with E gene qRT-PCR**.** B) Significantly higher C_t values in N gene qRT-PCR compared with E gene qRT-PCR (Wilcoxon matched-pairs signed rank test). C_t, cycle threshold; E, envelope; N, nucleocapsid; RAT, rapid antigen test.

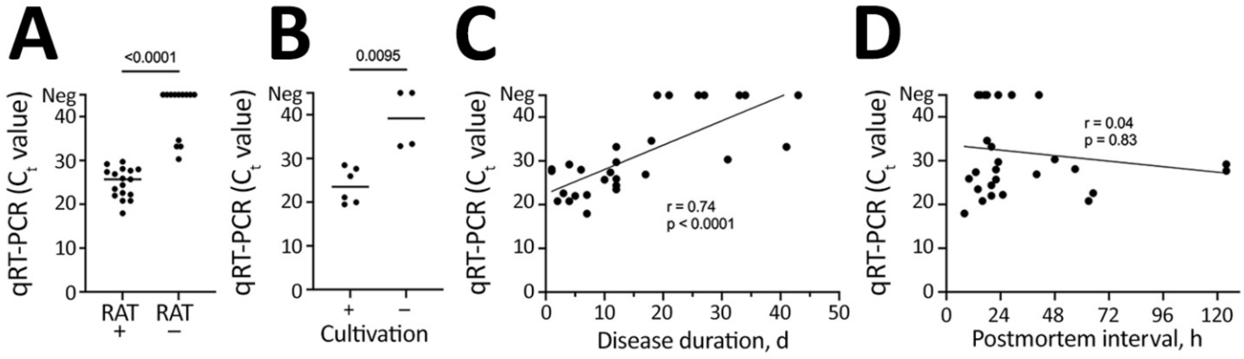

Appendix Figure 2. N gene qRT-PCR results. A) RAT negative cases show significantly higher C_t values compared with RAT positive cases (Mann-Whitney test). B) Cultivation negative and positive cases mirror C_t values of RAT results (Mann-Whitney test). C) Longer disease durations are significantly correlated with higher C_t values (Spearman correlation test). D) No significant correlation between postmortem intervals and C_t values (Mann-Whitney test). C_t, cycle threshold; E, envelope; N, nucleocapsid; RAT, rapid antigen test.

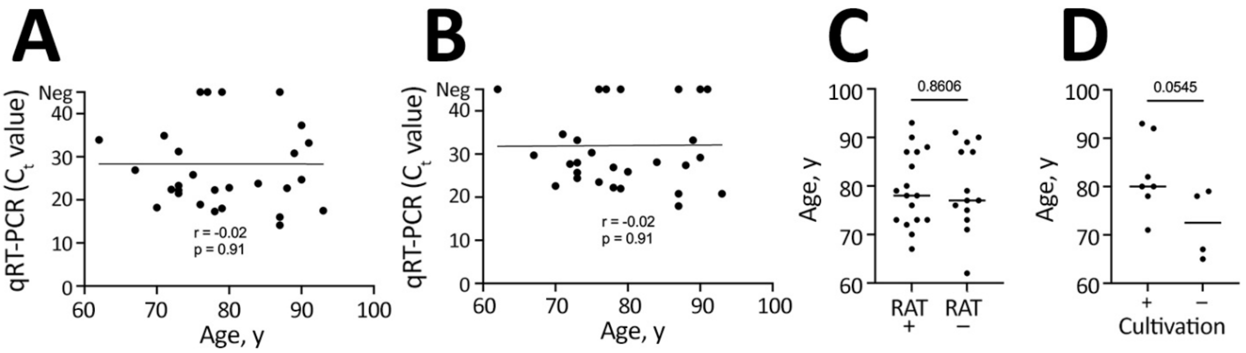

Appendix Figure 3. qRT-PCR, RAT, and cultivation results in relation to patient age. A–D) No significant correlation between age and C_t values (Spearman correlation test), RAT results (Mann-Whitney test), or cultivation results (Mann-Whitney test). C_t, cycle threshold; E, envelope; N, nucleocapsid; RAT, rapid antigen test.
